# Association of Metabolic Syndrome Components and Colonic Diverticulosis in the Very Elderly: A Tertiary Health Network Study

**DOI:** 10.7759/cureus.51610

**Published:** 2024-01-03

**Authors:** Hammad Liaquat, Farah Harmouch, Nishit Patel, Zarian Prenatt, Jill Stoltzfus, Berhanu Geme, Noel Martins, Kimberly Chaput

**Affiliations:** 1 Gastroenterology, St. Luke's University Health Network, Bethlehem, USA; 2 Internal Medicine, St. Luke's University Health Network, Bethlehem, USA; 3 Research Institute, St. Luke's University Health Network, Bethlehem, USA

**Keywords:** colon, diverticulosis, colonoscopy, elderly population, metabolic syndrome

## Abstract

Introduction

There is scarce data about the association of metabolic syndrome (MetS) or its components with the development of colonic diverticulosis (CD) in the elderly. We aim to determine the association of MetS and its components with CD in the elderly aged ≥75 years.

Methods

We conducted a retrospective chart review at St. Luke's University Health Network to identify patients who underwent a colonoscopy between 2011 and 2020. We collected data on patient demographics, comorbidities, and colonoscopy findings. Statistical analyses were conducted to compute means and frequencies of patient characteristics and rates of CD, as well as to test for associations between potential risk factors and the presence of CD.

Results

A total of 1239 patients were included with a median age of 80 years, 57.6% females, 89.5% Caucasians, 72.9% with CD, and 66.7% having a left-sided disease. On bivariate analysis, the older age group (p=0.02), Caucasian ethnicity (p=0.01), and hypertension (p=0.04) were found to be significant risk factors for developing CD. Multivariate regression analysis showed older age group and hypertension (OR=1.47, 95% CI: 1.66-2.02, p=0.02) were major risk factors. A significant proportion of patients with left-sided disease had Caucasian ethnicity (p<0.001), while female gender, obesity, and iron deficiency anemia were also seen more frequently, although without statistical significance.

Conclusion

In the elderly (>75 years old), our study found hypertension to be associated with an increased risk of CD, while impaired fasting glucose (IFG) was protective. Most patients exhibited isolated left-sided diverticulosis, with pan-diverticulosis associated with higher proportions of adverse health indicators, including American Society of Anesthesiologists (ASA) score ≥3, IFG, hypertriglyceridemia, hypertension, and hypothyroidism. Further research with larger sample sizes in similar age groups is needed to expand upon these findings.

## Introduction

Colonic diverticulosis (CD) is an age-related disease of the large bowel characterized by the existence of diverticula, which are small outpouchings that occur when the mucosa and muscularis mucosa herniate through the muscularis propria at locations where vasa recta penetrate the colon wall [1]. It is a common gastrointestinal disorder that has a significant burden on the quality of patient's lives as well as a burden on the health care system due to medical and surgical management [2]. While its burden on patient lives and the economy is high, little is known about the pathophysiology and risk factors that contribute to it [3]. The CD has the potential to develop along the entire length of the colon; however, studies have highlighted regional variations in its location and suggest that diet and genetics may impact the location as well [4-6]. Western industrialized countries like Europe, the USA, and Australia have a higher prevalence of distal CD, while Eastern countries like Asia tend to manifest proximal CD [1,7]. 

Age and geographic location have been identified as the two most important determinants of CD prevalence [8]. Since diverticulosis is typically asymptomatic and discovered incidentally, there is a lack of precise assessments regarding its true incidence and prevalence. As of 2016, the incidence of CD appears to be increasing worldwide, particularly in developed nations, where roughly two-thirds of adults over 18 years eventually develop this condition [9]. In the 20th century, it was reported that CD was found at rates of 2-10% [10]. More recent studies have indicated that the prevalence has increased to less than 10% in patients under the age of 40, about 50% in those ages 60 and above, and 50-66% in patients over the age of 80 [10]. Although many patients with CD are asymptomatic, this increase in the prevalence of diverticulosis has led to an increase in diverticular disease, including acute or chronic diverticulitis with or without complications, diverticular bleeds, and symptomatic diverticulosis [8]. 

CD is a burden on patients as well as the health care system. In 2002, the annual incidence of CD in the USA was about 300,000, with a prevalence of 2.5 million. It accounted for approximately 250,000 hospital visits, 1.5 million outpatient visits, and 3,372 deaths. In 2004, diverticular disease ranked as the third most common gastrointestinal hospital discharge diagnosis, the fourth most common reason for outpatient visits, and the fifth most costly gastrointestinal disease [11]. In the USA, 280,000 patients were hospitalized in 2009 for diverticular-related complications, with a financial burden of 2.7 billion [12]. According to a study conducted in 2016 by Peery et al., over 50% of individuals in the USA aged 60 and above are affected by CD [13].

Due to the financial burden and patient distress caused by CD, it is crucial for clinicians to identify patients at risk for diverticulosis and its complications in order to improve management in these individuals; this includes influencing modifiable risk factors such as environmental, behavioral, and dietary risk factors. In a recent study from Israel, metabolic syndrome (MetS) and its components have been of particular interest with their relationship to diverticulosis [14]. CD was found to be associated with age, male sex, hypothyroidism, and obesity, while diabetes mellitus was noted to be a protective factor. In contrast, a Japanese study found that hypertension and diabetes mellitus were found at a higher prevalence in patients with CD [15].

The aim of our study is to review patients over the age of 75 years who have undergone colonoscopy and to examine their co-morbidities, including MetS and its components, in order to identify risk factors for CD and its distribution within the colon.

## Materials and methods

Data collection 

The study was conducted at St. Luke's University Health Network. Retrospective chart reviews were performed on elderly patients (aged 75 years old and above) who have had a colonoscopy performed between 2011 and 2020. Exclusion criteria included incomplete colonoscopies and colonoscopy reports with missing data. Patient demographics, such as age, race, sex, and co-morbidities, were collected. Colonoscopy reports were reviewed, and abnormalities, including the presence of diverticulosis and location (right-sided, left-sided, and pan-diverticulosis), were noted. 

We have utilized the International Diabetes Foundation (IDF) definition for MetS [16], which is defined as the following: the presence of central obesity (BMI>30 kg/m^2^ if waist circumference is not available), along with any two of the following four factors: triglycerides >150 mg/dL, HDL <40 mg/dL in men or <50 mg/dL in women, impaired fasting glucose (IFG; Hba1c 5.7-6.4%), or diabetes (Hba1c >6.5) and/or hypertension (BP >130/85 mmHg).

Data analysis

To evaluate our primary outcome of diverticulosis, we conducted multivariable direct logistic regression to determine the independent contribution of age (75-84 years old versus 85 years old or greater) after adjusting for relevant demographic and clinical variables. Variables were recorded in tabular format, and statistical analyses were conducted. We used SPSS version 27 (IBM, Inc., Armonk, NY) to analyze our data, with a p-value <0.05 denoting statistical significance for all outcomes, without adjusting for multiple comparisons.

Prior to regression modeling, we conducted separate bivariate analyses using chi-square tests. Differences in frequencies between each variable were considered noteworthy if more than one percent difference in frequency was appreciated, while statistical significance was achieved only with a p-value <0.05. For the purpose of multivariate modeling, any comparisons with an associated p-value <0.20 were considered significant to be included in the regression analysis. All analysis outcomes from multivariate regression associated with a p-value <0.05 were considered significant. In addition to age, our potential covariates included gender, race (Caucasian versus non-Caucasian), American Society of Anesthesiologists (ASA) scores three and above, constipation, dementia, MetS, obesity, diabetes or pre-diabetes, hypertriglyceridemia, low high-density lipoprotein (HDL), hypertension, hypothyroidism, iron deficiency, and smoking status.

Additionally, we assessed for the presence of outliers and influential data points. There were only four out of 1,239 outliers (0.3%), and no influential data points were found based on the examination of normalized residuals, Cook's D, and leverage statistics. Therefore, we retained all patients in our analyses.

To ascertain model fit, we reported the omnibus chi-square statistic and the Hosmer-Lemeshow goodness-of-fit statistic. For each covariate, we present adjusted odds ratios (AOR) and 95% confidence intervals (CIs). 

As secondary outcomes, we assessed age-related differences in right-sided diverticulosis, left-sided diverticulosis, and pan-diverticulosis using separate chi-square tests. Additionally, we conducted analyses to assess differences among variables when comparing cohorts with left-sided disease and pan-diverticulosis. P-values <0.05 were considered statistically significant in this analysis, while any differences with a p-value <0.20 were included in the regression analysis. We did not conduct multivariable regression modeling for qualifying variables (race, MetS, and impaired fasting glucose), as age (our primary variable of interest/outcome) was not significantly different between the two groups following unadjusted comparisons. Furthermore, the race of patients was not amenable to multivariable modeling due to the small number of non-Whites in both outcome groups.

## Results

Data was collected on 1254 patients; however, there were only 1239 patients with complete data in our sample. The mean age was 80±4.95 years, with 1044 patients aged 75-84 years (84.3%) and 195 patients aged over 85 years (15.7%). There were 703 females (56.7%) and 536 males (43.3%). There were 1019 Caucasians (89.5%) and 130 non-Caucasians (10.5%). Among these patients, 903 (72.9%) had diverticulosis at any location within the colon. Specifically, 20 patients (2.2%) had a right-sided disease, 602 (66.7%) had a left-sided disease, and the remaining patients had pan-diverticulosis.

On bivariate analysis (Table 1), older age group (p=0.02), Caucasian race (p=0.01), and hypertension (p=0.04) were found to be significant risk factors for developing CD. A higher proportion of patients with a history of smoking cigarettes were at risk of developing CD (77 patients, 8.5%), which approached statistical significance (p=0.07). Additionally, a higher proportion of patients with CD had a history of constipation (317 patients, 35.1%, p=0.33), obesity (285 patients, 31.6%, p=0.36), and dementia (57 patients, 6.3%, p=0.10). IFG, hypertriglyceridemia, low HDL levels, and iron deficiency anemia were seen in a higher proportion of patients without diverticulosis but without any statistical significance.

**Table 1:** Bivariate comparisons of demographic and clinical variables for diverticulosis (n=1239) * Based on separate chi-square tests HDL - high-density lipoprotein

Characteristics (n, %)	Diverticulosis (n=903)	No diverticulosis (n=336)	p-value*
Age 75-84 years	748 (82.8%)	296 (88.1%)	0.02
Age ≥85 years	155 (17.2%)	40 (11.9%)	
Male gender	389 (43.1%)	147 (43.7%)	0.83
Caucasian ethnicity	820 (90.8%)	289 (86.01%)	0.01
Obesity	285 (31.6)	Yes: 97 (28.9%)	0.36
American Society of Anesthesiologists score >3	347 (38.4%)	132 (39.3%)	0.78
Constipation	317 (35.1%)	108 (32.1%)	0.33
Dementia	57 (6.3%)	13 (3.9%)	0.10
Metabolic syndrome	282 (31.2%)	106 (31.5%)	0.91
Impaired fasting glucose	311 (34.4%)	132 (39.3%)	0.11
Hypertriglyceridemia	150 (16.6%)	60 (17.9%)	0.60
Low HDL	168 (18.6%)	73 (21.7%)	0.22
Hypertension	748 (82.2%)	261 (77.7%)	0.04
Hypothyroidism	214 (23.7%)	77 (22.9%)	0.77
Iron-deficiency anemia	174 (19.3%)	69 (20.5%)	0.62
Current smoker or quit within 10 years of colonoscopy	77 (8.5%)	19 (5.7%)	0.07

Multivariate regression analysis showed that the older age group (OR=1.48, 95% CI: 1.05-2.16, p=0.04) and hypertension (OR=1.47, 95% CI: 1.66-2.02, p=0.02) continue to be major risk factors for developing CD (Table 2). 

**Table 2:** Multivariable logistic regression for diverticulosis (n=1239) * Omnibus chi-square p=0.001; Hosmer-Lemeshow goodness-of-fit p=0.71 AOR - adjusted odds ratio; CI - confidence interval

Characteristics	AOR (95% CI)*	p-value
Age >85 years (reference = 75-84 years)	1.48 (1.01-2.16)	0.04
Race (reference = White)	0.66 (0.45-0.98)	0.04
Dementia	1.68 (0.90-3.13)	0.10
Diabetes or pre-diabetes	0.80 (0.61-1.05)	0.11
Hypertension	1.47 (1.66-2.02)	0.02
Current smoker or quit within 10 years of colonoscopy	1.66 (0.99-2.80)	0.06

Next, we investigated the impact of age on the distribution of disease within the colon. No statistically significant results were found when comparing right-sided diverticulosis or left-sided diverticulosis to those patients without such distribution, respectively. However, the results for pan-diverticulosis approached significance in the higher age group (Table 3). As the sample of patients with isolated right-sided CD was too small for any meaningful comparisons, it was excluded from further analysis.

**Table 3:** Age-related comparisons for right-sided, left-sided, and pan-diverticulosis * Based on separate chi-square tests

Age (n, %)	Diverticulosis		p-value*
	Right-sided diverticulosis (n=20)	No right-sided diverticulosis (n=1219)	
Age 75-84	17 (1.6%)	1027 (98.4%)	0.93
Age >85	3 (1.5%)	192 (98.5%)	
	Left-sided diverticulosis (n=602)	No left-sided diverticulosis (n=637)	
Age 75-84	503 (48.2%)	541 (51.8%)	0.51
Age >85	99 (50.8%)	96 (49.2%)	
	Pan-diverticulosis (n=281)	No pan-diverticulosis (n=958)	
Age 75-84	228 (21.8%)	816 (78.2%)	0.1
Age >85	53 (27.2%)	142 (72.8%)	

Bivariate analysis was then performed on left-sided and pan-diverticulosis (Table 4). The majority of patients in this analysis were from the younger age group (82.6%), Caucasian (90.8%), and of female gender (56.8%), with a history of hypertension (82.8%) and left-sided diverticulosis (68.1%). A significant proportion of patients with left-sided disease were of Caucasian ethnicity (p<0.001), while female gender, obesity, and iron deficiency anemia were also observed more frequently in this cohort, although without statistical significance. 

**Table 4:** Bivariate comparisons of demographic and clinical variables for left-sided versus pan-diverticulosis (n=883) * Based on separate chi-square tests ASA - American Society of Anesthesiologists; IFG - impaired fasting glucose; HDL - high-density lipoprotein

Characteristics (n, %)	Left-sided diverticulosis (n=602)	Pan-diverticulosis (n=281)	p-value*
Age 75-84	503 (83.6%)	227 (80.8%)	0.36
Age >85	99 (16.4%)	54 (19.2%)	
Male gender	257 (42.7%)	124 (44.1%)	0.73
Female gender	345 (57.3%)	157 (55.9%)	
Race: White	562 (93.4%)	240 (85.4%)	<0.0001
Race: Non-White	40 (6.6%)	41 (14.6%)	
Obesity (BMI >30 kg/m^2^)	193 (32.1%)	85 (30.2%)	0.61
ASA >3	224 (37.2%)	116 (41.3%)	0.23
Constipation	211 (35.05%)	98 (34.9%)	0.99
Dementia	37 (6.1%)	18 (6.4%)	0.87
Metabolic syndrome	180 (29.9%)	97 (34.5%)	0.16
IFG	197 (32.7%)	107 (38.1%)	0.11
Hypertriglyceridemia	99 (16.4%)	50 (17.8%)	0.60
Low HDL	114 (18.9%)	51 (18.1%)	0.80
Hypertension	495 (82.2%)	237 (84.3%)	0.37
Hypothyroidism	134 (22.3%)	72 (25.6%)	0.26
Iron-deficiency anemia	118 (19.6%)	51 (18.1%)	0.63
Current smoker or quit within 10 years of colonoscopy	52 (8.6%)	24 (8.5%)	0.97

## Discussion

Diverticulosis of the colon is the most frequent finding seen during colonoscopy [7,17]. It has been found in 71.4% of patients above 80 years of age in a prior study [18], which is comparable to our results. Ethnicity can also play a role in the severity and distribution of CD. A 2011 study found that Black patients had a 3.54 times higher risk of developing right-sided CD compared to Caucasians [19]. The sample of non-Caucasian patients was small compared to Caucasian patients, which was comparable to our results. Additionally, we found Caucasians have a significantly higher proportion of left-sided CD compared to other races. 

MetS has also been associated with several gastrointestinal and hepatobiliary pathologies. In one study, MetS was strongly associated with patients who had erosive esophagitis and Barrett's esophagus [20]. The incidence of MetS was noted to be 30.9% in patients with acute pancreatitis in a Chinese case-control study involving 705 patients [21]. MetS has long been associated with non-alcoholic fatty liver disease (now referred to as metabolic dysfunction-associated steatotic liver disease), which is an emerging risk factor for hepatocellular carcinoma [22]. Colorectal cancer was also found to be significantly associated with MetS in various populations [23,24]. 

Of particular interest are MetS and its components, such as hypertension, obesity, IFG, and dyslipidemia, including low HDL and elevated triglycerides per the International Diabetes Foundation guidelines [15]. Recent literature has focused on exploring the association between MetS and CD. Yan et al. discovered risk factors, such as age over 60 years, obesity, hypertension, and hypertriglyceridemia, were associated with an increased risk of CD [25]. Teixeira et al. found bidirectional links between CD and MetS, with central obesity, hypertension, and insulin resistance correlating individually with CD risk [26]. Bae et al. highlighted that MetS is strongly associated with asymptomatic CD risk, particularly in those with a larger waist circumference and BMI [27]. Ojemolon et al. conducted a decade-long joinpoint analysis investigating the trends in hospitalizations for colonic diverticular disease in patients with morbid obesity and found that metabolic factors associated with morbid obesity may contribute to the development of CD and its related hospitalizations [28]. While these studies collectively provide insights into the possible link between MetS and CD, further investigation is warranted to fully elucidate the underlying mechanisms and establish the clinical implications of this relationship, particularly within the aging population. Comparatively, our current study focuses specifically on advanced age (≥75 years), aiming to provide a more nuanced understanding of how MetS interplays with the occurrence of CD in this particular demographic. 

The bivariate analysis conducted in our study revealed a significant association of CD with advanced age (>85 years old), Caucasian race, and hypertension, while the history of dementia, being a current or recent smoker, as well as the absence of IFG approached significance. Multivariate regression analysis confirmed that older age, race, and hypertension are significant risk factors for CD. Our study had similar findings to a study conducted by Kopylov et al. [14] on an Israeli population, which showed the presence of diverticulosis significantly associated with increasing age, obesity, hypothyroidism, and the absence of diabetes. The study by Sakuta et al. [15], performed on a Japanese population, revealed an increased association between the presence of type 2 diabetes mellitus (unlike our study) and hypertension (similar to our findings).

We also examined risk factors that may contribute to left-sided and pan-diverticulosis. Due to the small number of patients with isolated right-sided diverticulosis (n=5) compared to the rest of our study population, they were excluded from our study. The small sample size is not surprising, as right-sided diverticulosis is more prevalent in Eastern countries - a difference that is partly due to genetic and environmental factors [5,29]. The majority of our patients were noted to have left-sided diverticulosis only (69.4%). Caucasians and obese patients were more likely to have left-sided diverticulosis in our study, while a higher proportion of patients with CD spreading throughout the colon belonged to the higher age group (p=0.10). Studies in Japan did reveal that diverticulosis started in the right colon prior to involving the rest of the colon [30]. Hjern et al. studied a population of immigrants in Sweden from Eastern countries as well as the West [31]. Their findings suggested an increase in diverticular disease in the Eastern population after a short period of acculturation in Sweden. This may be partially due to a difference in diet rich in red meats and less fiber intake. Furthermore, a study performed in Korea, which compared Buddhist priests (who follow a vegetarian diet) to patients on a regular diet, revealed that male gender, regular diet, and BMI >25 kg/m^2^ were associated with an increased risk of right-sided CD. On the other hand, left-sided diverticulosis was associated with a regular diet but not elevated BMI [32]. Dietary fiber intake may decrease the incidence of CD through reduced colonic inflammation, lower level of contractions needed to manipulate the stool, reduced subsequent herniation in the colonic wall, and alterations in the intestinal microbiome, but such associations have been met with conflicting results [33,34]. Another study of 2164 patients highlighted that alcohol consumption and smoking are significant risk factors for CD, particularly for right-sided or pan-diverticular disease, in the Japanese population [35]. Research in Caucasian populations has shown similar associations [36,37]. The role of obesity in CD is unclear. Visceral adiposity (VAT) rather than overall elevated BMI has been significantly associated with left-sided CD in past literature [38,39]. VAT is associated with higher levels of pro-inflammatory leptin and lower levels of the anti-inflammatory mediator adiponectin, but data about the association of this imbalance with the development of CD is evolving [40]. 

To the best of our knowledge, this study is one of the first in the USA to evaluate the relationship between MetS, its components, and the presence of CD, including its location in the colon, within the very elderly age groups. Given the higher likelihood of comorbidities in this age group, diagnosis of CD is important to maintaining or improving the quality of life in patients aged >75 years old. Diagnosing CD in this population is also important for preventing complications, such as diverticulitis, perforation, or bleeding, which can lead to severe outcomes in this age group. Our study provides avenues for future research in the elderly age groups with CD and highlights risk factors that may influence the development, distribution, and severity of the condition, which can be explored in subsequent investigations. Colonoscopy as a means to diagnose CD has been compared to other modalities in the past. Double-contrast barium enemas are superior in diagnosing CD when compared to colonoscopy. The left colon has a narrower lumen that can decrease the field of view during colonoscopy, which is one factor that can decrease CD diagnosis yield from colonoscopy. At the same time, colonoscopy is the best test to detect small polyps and cancer that may occur in CD sites [41]. Cross-sectional imaging can help diagnose complicated CD, which may be missed on colonoscopy as well [42]. This could be a limitation of our study as we only selected patients in whom CD was discovered on colonoscopy. Therefore, it is possible that some patients with CD may have been overlooked. Nonetheless, as the criteria for inclusion/exclusion were consistently applied across the sample, this may help mitigate some of the impact of this limitation. Furthermore, the sample sizes were not uniform when patients were divided into age groups, by presence or absence of CD, or even when comparing different distributions of the disease. This discrepancy is an inherent limitation in retrospective studies.

## Conclusions

Our study revealed that in an elderly population (>75 years old), hypertension was associated with an increased risk of developing CD, while IFG was protective and associated with a decreased risk. In terms of the location of diverticulosis, the majority of our patients were found to have isolated left-sided diverticulosis. Although there were no significant variables when comparing left-sided and pan-diverticulosis, we noted that pan-diverticulosis had a higher proportion of patients with ASA ≥3, IFG, hypertriglyceridemia, hypertension, and hypothyroidism, while left-sided diverticulosis had a higher proportion of obese patients. Additional research in such age groups with larger sample sizes is needed to investigate our findings further.
